# Detection of circulating tumour cell clusters in human glioblastoma

**DOI:** 10.1038/s41416-018-0186-7

**Published:** 2018-08-01

**Authors:** Ilona Krol, Francesc Castro-Giner, Martina Maurer, Sofia Gkountela, Barbara Maria Szczerba, Ramona Scherrer, Niamh Coleman, Suzanne Carreira, Felix Bachmann, Stephanie Anderson, Marc Engelhardt, Heidi Lane, Thomas Ronald Jeffry Evans, Ruth Plummer, Rebecca Kristeleit, Juanita Lopez, Nicola Aceto

**Affiliations:** 1 Department of Biomedicine, Cancer Metastasis Laboratory, University of Basel and University Hospital Basel, CH-4058 Basel, Switzerland; 2 Swiss Institute of Bioinformatics, Lausanne, Switzerland; 3 Basilea Pharmaceutica International Ltd., CH-4058 Basel, Switzerland; 4 Drug Development Unit, The Royal Marsden Hospital and The Institute of Cancer Research, London, UK; 5 Institute of Cancer Sciences, University of Glasgow, Glasgow, UK; 6 Northern Centre for Cancer Care, Newcastle upon Tyne, UK; 7 University College London Cancer Institute, London, UK

**Keywords:** CNS cancer, Cancer genomics

## Abstract

Human glioblastoma (GBM) is a highly aggressive, invasive and hypervascularised malignant brain cancer. Individual circulating tumour cells (CTCs) are sporadically found in GBM patients, yet it is unclear whether multicellular CTC clusters are generated in this disease and whether they can bypass the physical hurdle of the blood–brain barrier.  Here, we assessed CTC presence and composition at multiple time points in 13 patients with progressing GBM during an open-label phase 1/2a study with the microtubule inhibitor BAL101553. We observe CTC clusters ranging from 2 to 23 cells and present at multiple sampling time points in a GBM patient with pleomorphism and extensive necrosis, throughout disease progression. Exome sequencing of GBM CTC clusters highlights variants in 58 cancer-associated genes including *ATM*, *PMS2*, *POLE*, *APC*, *XPO1*, *TFRC*, *JAK2*, *ERBB4* and *ALK*. Together, our findings represent the first evidence of the presence of CTC clusters in GBM.

## Introduction

Human glioblastoma (GBM) is the most common and aggressive primary brain cancer in adults.^1^ Yet, despite its characteristic invasive features and hypervascularity determined by angiogenic recruitment, only 0.4–2% of GBM patients develop metastasis outside of the central nervous system.^2^ The rarity of these metastatic events has been attributed to the short-term survival of patients after initial GBM diagnosis, leaving insufficient time for establishment of extracranial lesions, as well as to the presence of the blood–brain barrier, which physically separates the brain from the rest of the body.^3^ Circulating tumour cells (CTCs) are cancer cells that detach from a primary tumour lesion or a metastatic deposit and enter the bloodstream.^4^ Although individual CTCs have been sporadically observed in GBM,^5–8^ it is unclear whether multicellular CTC clusters^9^ are generated and are able to pass through the blood-brain barrier in patients with GBM.

## Materials and methods

### Patient selection and recruitment

Patients were participants in the GBM arm of the ongoing study ‘An open-label Phase 1/2a study of oral BAL101553 in adult patients with advanced solid tumours and in adult patients with recurrent or progressive glioblastoma or high-grade glioma’ (NCT02490800). The main eligibility criteria for GBM patients in the study are measurable disease, defined by contrast-enhancing magnetic resonance imaging (MRI), and an Eastern Cooperative Oncology Group performance status of ≤ 2. The study was conducted in accordance with the Declaration of Helsinki (2000) and the International Conference on Harmonisation Guidelines for Good Clinical Practice. The study was approved by each study centre’s Research Ethics Committee and all patients provided written informed consent before enrolment.

### Mouse experiments

All mouse experiments were carried out according to institutional and cantonal guidelines (approved mouse protocol 2781, cantonal veterinary office of Basel-City). To measure GBM CTC intravascular aggregation, Nod Scid Gamma mice (The Jackson Laboratory, Bar Harbor, Maine, USA) were injected through the tail vein with a 1:1 mixture of T98G-GFP and T98G-RFP GBM cells, and upon injection, whole blood was withdrawn through a heart puncture and processed with the Parsortix device to characterise CTC composition.

### GBM CTC enumeration

To test the GBM cell capture rate, healthy donor blood samples were drawn in three different collection tubes: CF DNA blood collection tube (BCT) (Streck, 218997), CellSave (CellSearch, 7900005) and Cyto-Chex BCT (Streck, 213386). The blood was then spiked with 300 human GBM T98G cells (Sigma, 92090213, CRL-1690) stably expressing green fluorescent protein (GFP). T98G-GFP cells were generated by transduction with UBC-GFP-T2A-Luc-expressing lentiviral particles (SBI, BLIV201PA-1-SBI). After incubation at room temperature (RT) for 24, 48, 72, and 96 h, samples were processed with the Parsortix microfluidic system for CTC enrichment. After wash with phosphate-buffered saline (PBS), Parsortix cassettes were scanned and GFP-positive cells were counted using Leica DMI 6000 microscope. To test artificial clustering of CTCs within the Parsortix cassettes, we spiked 100 single T98G-GFP and 100 single T98G-RFP into CF DNA BCT (Streck, 218997) tubes containing healthy donor blood. Upon processing through the Parsortix microfluidic system, we enumerated the ratios between single colour CTC and multicolour CTCs as a direct measure of on-chip CTC aggregation.

### Tumour volume measurements

The enhancing tumour volume (cm^3^) and the corresponding FLAIR signal abnormality volume (cm^3^) in patients were calculated with Horos, an open source medical image viewer (LGPL license at Horosproject.org, sponsored by Nimble Co LLC d/b/a Purview in Annapolis, MD US).

### CTC capture from GBM patients

Patients’ blood samples were collected into the Streck CF DNA BCTs (Streck, 218997) and directly shipped at controlled temperature (RT). Samples were then loaded into the Parsortix microfluidic device within 24–48 h after collection from the patient and immediately processed for CTC enrichment. CTCs were then enriched from blood samples at 16 °C in disposable Parsortix cassettes (GEN3D6.5, ANGLE) according to the manufacturer’s instruction and with a customised processing flow rate of 0.33 mm/s in the narrowest cassette gap (6.5 μm). After separation, GBM cells within cassettes were washed with PBS and further processed for immunostaining with antibodies against EGFR (Cell Signaling), Ki67 (Sigma), EB1 (kindly provided by Basilea Pharmaceutica), and CD45 (Thermo Fischer). As an additional confirmation, putative CTCs as well as a matched primary tumour biopsy of patient 4 were stained with antibodies against SOX2 (Cell Signaling).

### Micromanipulation and exome sequencing

CTCs were released from the Parsortix cassette into ultra-low attachments culture plates. Cells were picked using micromanipulation (CellCelector, ALS) and transferred to RTL Plus buffer (Qiagen) within individual microcentrifuge tubes. Next, genomic DNA was amplified using the MDA method (GenomiPhi V3, GE Healthcare) and subject to Illumina library preparation with SureSelect XT Human All Exon V6 + Cosmic kit (Agilent Technologies). Sequencing was performed on HiSeq 2500 platform (Illumina) with 101 bp paired-end mode. For primary tumour sequencing, three 10 μm-thick paraffin sections from a primary tumour biospy were digested in DNA digestion buffer (50 mM Tris-HCl pH 8.5, 1 mM EDTA pH 8.0 and 0.5% Tween with addition of Proteinase K) in an eppendorf tube at 56 °C for 1 h, followed by 1 h incubation at 90 °C and 5 min at 95 °C. Samples were then kept on ice until RNase A treatment was performed (30 minutes at 37 °C). Next, DNA was precipitated using 7.5 M ammonium acetate and 100% isopropanol. After washing with 70% ethanol, the DNA pellet was air dried and then resuspended in standard Tris-containing buffer. Sequencing was performed on HiSeq 2500 platform (Illumina).

### Exome-sequencing data analysis and mutation calling

After quality control performed with FastQC (v0.11.4), reads were mapped to the GRCh38 human reference genome using BWA-mem (v0.7.13) algorithm and sorted using Samtools (v1.3.1). Reads were then processed using Picard MarkDuplicates (v2.9.0) to remove duplicated reads and realigned using GATK IndelRealignment (v3.7.0) to improve alignment accuracy around indels. We used the interactive platform Ginkgo (http://qb.cshl.edu/ginkgo) to compute and plot the copy number profiles of single-cells. Input bed files were generated from BAM alignments using BEDTools (v2.26.0) and genomic coordinates were converted from GRCh38 to GRCh37 UCSC liftOver tool. Ginkgo was run with a using variable bin size of 500 kb simulated with 101 bp with BWA and normalised read counts for cell segmentation. Variants were identified using Monovar (20160514 update) for low-input DNA samples and bcftools (version 1.6) for primary GBM of patient 4. Resulting variants were annotated using snpEff (v4.3p) with Ensembl GRCh38.86 gene models. Putative somatic SNV were defined as variants not present in the white blood cell (WBC) pool and whose frequency in the dbSNP (build 150) was < 1%. In addition, a more stringent set of somatic single nucleotide variants (SNVs) was obtained by filtering out loci that were covered with < 5 reads in the WBC pool. Candidate somatic driver variants were defined as variants annotated with high or moderate impact according to snpEff, previously reported in the COSMIC (version 81) or located in recurrently mutated genes in gliobastoma according to the CGC database (version 81). Data analysis was conducted in R (version 3.4.0). Data visualisation was performed with the R package UpSetR and the Bioconductor package GenVisR.

## Results

### CTC detection strategy in GBM patients

**Fig. 1:**
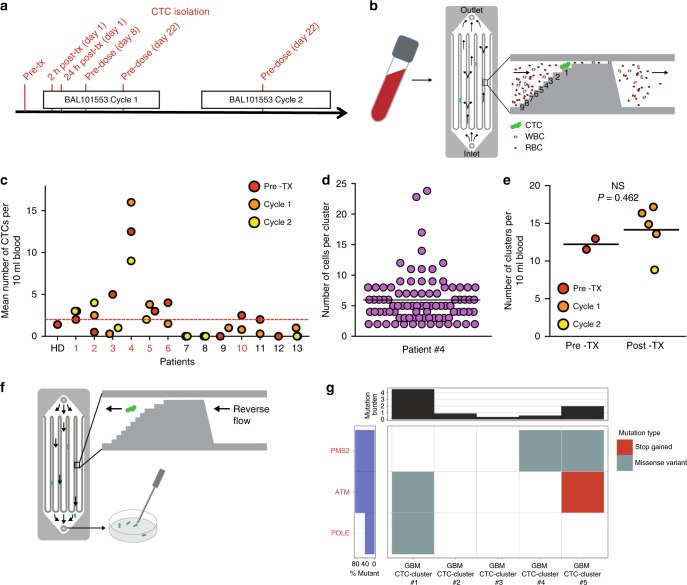
Identification of GBM CTC clusters. **a** Schematic overview of the CTC isolation strategy (red) in GBM patients undergoing treatment with BAL101553. Each patient underwent several blood draws: one or two blood draws before BAL101553 treatment (pre-tx), four during the first BAL101553 cycle and one during the second BAL101553 cycle. **b** Schematic representation of our CTC enrichment strategy with the Parsortix microfluidic device. CTCs are captured with a size-based antigen-agnostic approach, while red and white blood cells flow through the device. **c** Plot showing the mean number of CTCs found at each sampling time point (pre-treatment: pre-TX; cycle 1 and cycle 2) in 13 GBM patients. Healthy donors samples (HD; *n* = 3) provide a false-positive threshold of two putative CTCs per 10 ml blood. Patients with > 2 putative CTCs per 10 ml of blood during at least one time point are shown in red. **d** Plot showing the number of cells per each GBM CTC cluster isolated from patient 4. **e** Plot showing the number of GBM CTC clusters per 10 ml of blood identified in patient 4 during each blood draw, both pre-treatment (pre-TX) and post-treatment (cycle 1 and cycle 2) with BAL101553. *P* = 0.462 by Student’s *t*-Test. NS: not significant. **f** Schematic representation of the CTC isolation strategy by flow reversion and picking with a micromanipulator. **g** Putative somatic mutations in glioblastoma candidate driver genes found in GBM CTC clusters. The central panel shows the mutated genes coloured by the predicted consequence of the mutations. The top panel shows the number of mutations per sample and the left panel shows the percentage of samples that have a mutation in each represented gene

Patients selected for CTCs investigation were part of an ongoing open-label Phase 1/2a study arm of oral BAL101553, a water-soluble microtubule inhibitor, in adult individuals with recurrent or progressive GBM or high-grade glioma. Upon informed consent, serial peripheral blood samples were drawn in Streck tubes from 13 GBM patients at time points related to BAL101553 administration, including (1) within the 2 weeks before the first study-drug treatment; (2) pre-dose, (3) 2 h post-dose and (4) 24 h post-dose on Cycle 1 Day 1; and pre-dose on (5) Cycle 1 Day 8, (6) Cycle 1 Day 22 and (7) Cycle 2 Day 22 (Fig. 1a). Blood samples were then processed within 48 h for CTC enumeration and characterisation. To investigate CTC number and composition in GBM patients, we made use of a commercially available CTC detection method, the Parsortix microfluidic technology, customised to detect both single and clustered GBM CTCs in an antigen-independent manner and at > 98% capture efficiency, up to 48 h upon fixation in Streck CF DNA tubes (see methods and Supplementary Fig. 1a-c), and without the occurrence of artificial CTC aggregates during processing (Supplementary Fig. 1d,e). Briefly, blood samples were run through the Parsortix microfluidic cassette at a flow rate corresponding to 0.33 ml/s in the narrowest channel section, ensuring label-free physical capture of CTCs and their separation from blood components (Fig. 1b). Upon capture, CTCs were stained with a dedicated GBM CTC antibody cocktail containing antibodies against EGFR, Ki67 and the microtubule-associated protein EB1, as well as against the WBC marker CD45 to exclude leukocyte contamination (Supplementary Fig. 2). Putative GBM CTCs were scored positive when corresponding to at least one of these criteria: (1) cell diameter of at least 9 μm and negative CD45 staining, and/or (2) positive EGFR, Ki67 or EB1 staining and negative CD45 staining. With these parameters, and using healthy blood donors (*n* = 3) to define a false-positive detection threshold, we found that 7/13 patients were positive for at least 3 putative CTCs per 10 ml of blood during at least 1 time point (Fig. 1c). Importantly, radiological imaging determined that none of the 13 patients enroled in the study developed extracranial metastasis (data not shown), and no association was observed between functional MRI volume and the presence of CTCs, most likely due to the small patient cohort (*n* = 13) (Supplementary Fig. 3).

### Detection of GBM CTC clusters

Among patients that resulted positive for putative GBM CTCs, we focused on a 43-year-old female patient (4), featuring the highest number of CTC events in the peripheral circulation. The patient had a first surgical resection of a right parietal GBM, which showed a high-grade astrocytic tumour with pleomorphism, mitoses, extensive necrosis and prominent vascular proliferation. Following surgery, the patient underwent chemoradiotherapy comprising standard radiation plus concomitant and subsequent adjuvant temozolomide. Imaging at the end of adjuvant treatment suggested progressive disease, requiring a second surgical resection followed by procarbazine/lomustine/vincristine (PCV) chemotherapy and re-irradiation. Further disease progression was noted and subsequently the patient was enroled onto the BAL101553 clinical trial (Supplementary Fig. 4a). MRI of the brain before commencing BAL101553 therapy and CTC enumeration showed enhancing tumour areas in the posterior aspect of the right parietal lobe, infiltrating the corpus callosum and extending periventricular along the occipital horn of the right lateral ventricle and inferiorly into the temporal lobe (Supplementary Fig. 4b). There were no other abnormalities seen on baseline whole-body radiological imaging (data not shown). CTC analysis revealed the presence of clusters of putative CTCs in the blood of this patient, ranging from 2 to 23 cells and expressing various combinations of EGFR, Ki67 and EB1 markers, yet negative for CD45 (Fig. 1d and Supplementary Fig. 4c). As a further validation step, putative GBM CTC clusters, as well as a biopsy from the primary GBM of patient 4 were stained with the GBM marker SOX2, revealing SOX2-positive cells in both specimens (Supplementary Fig. 4d, e). Similar to breast cancer,^9^ we also confirmed that GBM CTCs are not able to form intravascular aggregates upon injection in the venous circulation of mice (Supplementary Fig. 5a,b), indicating that the putative CTC clusters observed in patient 4 are likely to be direct derivatives of the primary GBM tumour. As a relatively stable number of putative CTC clusters were observed in the samples taken prior to and after initiation of BAL101553 therapy, it does not appear as though the treatment itself impacted on CTC clusters release at this dose level (Fig. 1e).

### Exome sequencing of GBM CTC clusters

To assess whether the cell clusters isolated from the patient of interest were bona fide GBM cells, we released them in solution from the Parsortix device and, with a semi-automatic micromanipulator, we deposited each cluster individually into lysis buffer (Fig. 1f), followed by DNA amplification and library preparation for exome sequencing. Six samples were successfully sequenced, including one WBC pool as germline control and five putative GBM CTC clusters. We obtained an average of 41 million reads per sample, corresponding to a median coverage depth of 32.6 × and an average breadth of 18.7%, in range with typical performance of single-cell-resolution exome sequencing of fixed cells^10^ (Supplementary Fig. 6a and Suppl. Table 1). To address whether our putative GBM CTC clusters were indeed of cancer origin, we adopted two parallel approaches. First, we inferred DNA copy number variations (CNVs) from exome-sequencing data in all our samples (see Methods section). In general, while CNV assessment from single-cell-resolution exome sequencing cannot precisely determine CNV in exact regions due to the relative low coverage and possible amplification biases, we sought to use it to investigate general aneuploidy trends across cells. We found that while predicted ploidy for WBC control pool was 2, all putative GBM CTC clusters displayed a predicted ploidy ranging from 2.2 to 4.1, suggesting a higher degree of genomic rearrangements compared with the WBC control pool (Supplementary Fig. 6b). Second, we identified variations at the nucleotide level in both the WBC control pool as well as the putative GBM CTC clusters. Genetic variations identified in both the putative CTC clusters and the WBC control pool were considered likely to be part of the patient’s germline variants and not included for further analyses. We then interrogated specific GBM-associated genes (see Methods section) and found among them a premature STOP codon in *ATM* gene and missense variants in *PMS2* and *POLE* genes in GBM CTC clusters (Fig. 1g and Suppl. Table 2). Of note, the region corresponding to the STOP codon in *ATM* gene and the majority of the other variants were covered and found to contain a wild-type sequence in the WBC pool control sample (Suppl. Table 2). More generally, when interrogating the whole exome of the putative GBM CTC clusters, we found that they were carrying at total of 116 variants (possibly both driver and passenger mutations) comprised in a total of 58 cancer-associated genes (i.e., genes that were reported in the COSMIC database and whose corresponding substitutions were leading to either structural variants, premature STOP codon, or missense variants) including *APC*, *XPO1*, *TFRC*, *JAK2*, *BRCA2*, *ERBB4* and *ALK*, in addition to the GBM-associated genes *ATM*, *PMS2* and *POLE* (Supplementary Fig. 6c and Suppl. Table 3). Further, despite the notorious difficulties in capturing the entire mutational heterogeneity of an individual’s primary GBM with a single biopsy,^11^ we asked whether any of the mutations found at the level of GBM CTC clusters were also detectable in the primary tumour. Deep sequencing of a matched primary tumour biopsy of patient #4 revealed that 16 out of 116 GBM CTC cluster mutations were indeed also found in the primary tumour (Suppl. Table 3), including mutations in *POLE*, *BRCA2*, *ALK*, and *TFRC* (Suppl. Table 4). Importantly, several of these loci that contained mutations both in the primary tumour and GBM CTC clusters were covered and found to contain a wild-type sequence in the WBC pool control sample (Suppl. Table 4), conclusively demonstrating the GBM origin of the isolated CTC clusters.

Together, our results provide the first evidence that GBM can release clustered CTCs, characterised by a distinct mutational profile, which pass beyond the blood–brain barrier and reach the peripheral circulation.

## Discussion

Although CTC clusters have been highlighted as highly-efficient metastatic precursors,^9^ they remain a poorly characterised feature of the metastatic process, mainly due to their dilution factor in the blood of patients and challenges to isolate them without disrupting cell–cell interactions. As further confounding factors, physiological features such as the localisation of the primary tumour, circulation dynamics and entrapment in capillary beds may strongly influence the number of detected CTC clusters in the peripheral circulation of a given patient.

Our work provides the first evidence that circulating glioblastoma clusters can overcome the blood–brain barrier and reach the peripheral circulation. Yet, much remains to be understood in regard to their biology and clinical relevance in this disease, possibly using larger patient cohorts. In the future, it will be of interest to explore the transcriptome of CTC clusters, to define their genetic heterogeneity as compared with the primary tumour, and to assess whether or not their presence in the peripheral circulation correlates with GBM aggressiveness. We speculate that since cancer cells are able to enter the blood circulation as clusters in a non-epithelial cancer such as GBM, a limited cell–cell junction repertoire^12–14^ may still be sufficient to sustain CTC clustering and ensure the preservation of multicellular structures beyond vascular barriers. Further studies will be needed to test this hypothesis and to identify key factors involved in CTC cluster generation and passage through physical barriers. In addition, the identification of the essential cell–cell junction requirements to maintain CTC clustering may lead to the development of novel cluster-targeting agents.

## Electronic supplementary material

Supplementary Figure 1

Supplementary Figure 2

Supplementary Figure 3

Supplementary Figure 4

Supplementary Figure 5

Supplementary Figure 6

Supplementary Table 1

Supplementary Table 2

Supplementary Table 3

Supplementary Table 4

## **A**vailability of data and material

Exome-sequencing data have been deposited to the European Nucleotide Archive (ENA, EMBL-EBI) with the accession number PRJEB27438.
